# Pain, Financial Hardship, and Employment in Cancer Survivors

**DOI:** 10.1200/JCO.21.01812

**Published:** 2021-11-18

**Authors:** Victoria S. Blinder

**Affiliations:** ^1^ Memorial Sloan Kettering Cancer Center, New York, NY

More than half of working-age cancer survivors (age 18-64 years) in the United States have experienced cancer-associated financial hardship, such as accumulating debt, filing for bankruptcy, worrying about their financial stability, or delaying or foregoing medical care because of concerns about cost.^1^ Not surprisingly, loss of employment and decreased earnings, which may result from working less hours or taking unpaid leave, are associated with greater risk and severity of financial hardship.^1,2^

**THE TAKEAWAY**

In the article that accompanies this editorial, Halpern et al^3^ found that, among cancer survivors, ongoing pain is associated with decreased work, including retiring early, feeling less productive at work, and changing to a part-time or otherwise less demanding job. These findings indicate that there is an unmet need for improved access to adequate and free or affordable pain management among cancer survivors.

**FIG 1.**
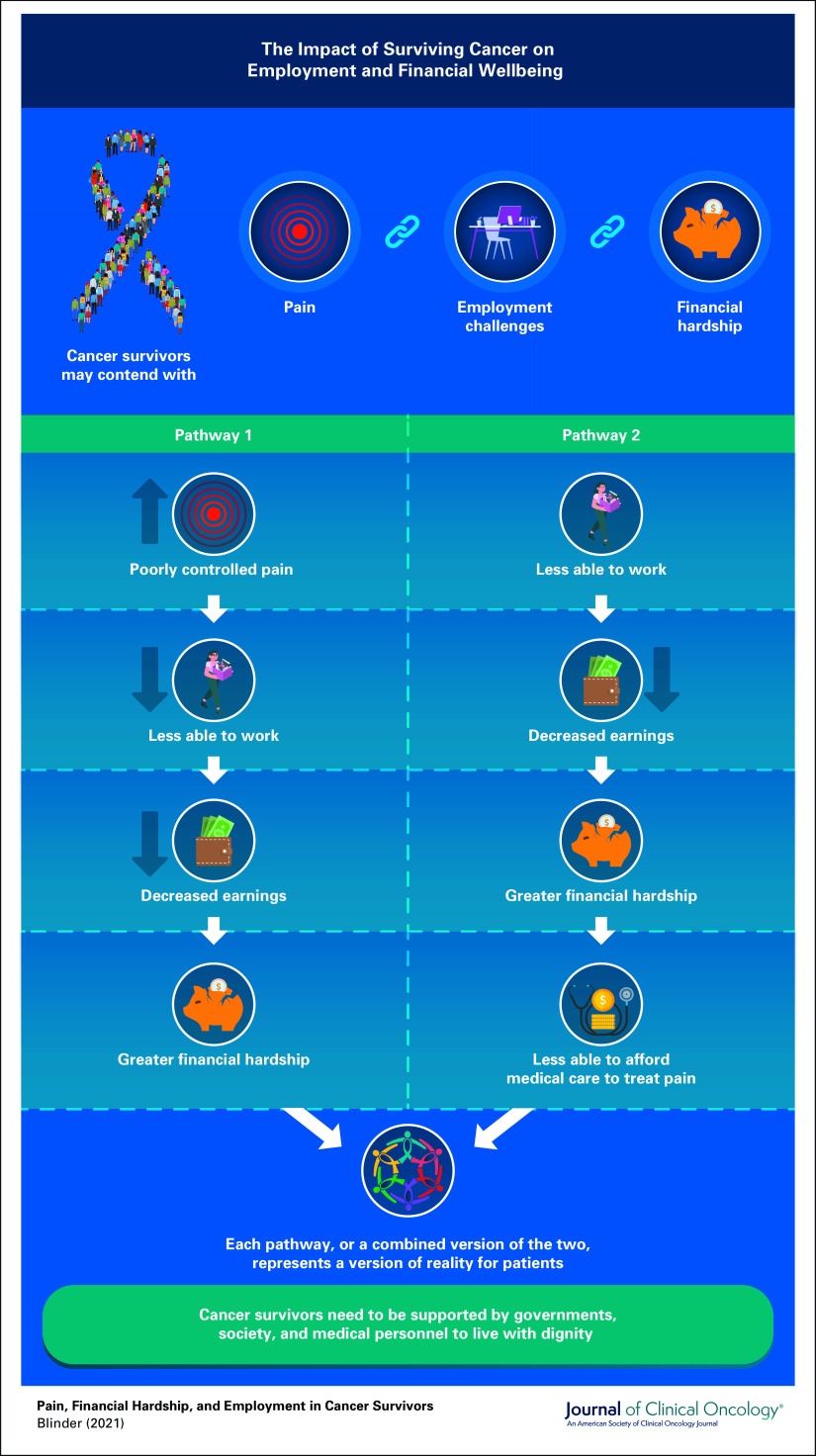
The impact of surviving cancer on employment and financial well-being.

In the article accompanying this editorial, Halpern et al^3^ suggest that improving pain management among cancer survivors could be a solution. Using data from a nationally representative sample of cancer survivors, Halpern et al^3^ found a relationship between pain, employment, and financial outcomes. Specifically, they identified a dose-response relationship between the level of pain and retiring early, feeling less productive at work, and changing to a part-time or otherwise less demanding job. They also identified a dose-response relationship between pain and cost-related delays in filling of a prescription or seeking medical care, defined as a visit for treatment or to see a specialist. Although these findings are based on a cross-sectional survey, and the exact causal relationships between these outcomes have not been described, we may infer two potential inter-related pathways linking pain, financial hardship, and employment (Fig 1). In one pathway, patients with cancer who have poorly controlled pain are less able to work, have decreased earnings, and, therefore, suffer greater financial hardship. In the other pathway, patients with cancer who are less able to work have decreased earnings, suffer greater financial hardship, and are, therefore, less able to afford the cost of medical care to adequately treat their pain. It is likely that each pathway, or a combined version of the two, represents a version of reality for patients and each represents a moral shortcoming for our society. Importantly, both pathways also present us with solutions that we may implement as a society, as clinicians, and as researchers to better care for patients with cancer.

As a society, we must do better to ensure that people have access to high-quality medical care, regardless of their ability to pay, without risking financial hardship. The study accompanying this editorial, as well as others previously published, has linked financial hardship to delayed or inadequate care (eg, medication nonadherence) and to symptom burden.^4,5^ This is a pervasive and, among wealthy nations, a uniquely American problem. In a 2017 report, the Commonwealth Fund found that US patients had the highest cost exposure among 10 high-income countries.^6^ At the policy level, the United States would do well to adopt some of the measures that other high-income countries use to improve access to health care, such as government-mandated caps of out-of-pocket spending for all health care (including prescription drugs) and cost-sharing exemptions for low-income and/or chronically ill patients. Additionally, we should improve access to nonpharmacologic pain management options, such as physical therapy and acupuncture, which are inconsistently covered by health insurance plans.^7^

As clinicians, in addition to advocating for policy changes that would improve our patients' access to care, we must advocate for our individual patients. To ensure that our patients who experience pain have access to adequate treatment, we should follow the guidelines from the American Society of Clinical Oncology that tells us to screen our patients for pain at every clinical encounter.^8^ When writing a prescription for pain medication, we should check to make sure that patients are financially able to fill it and to take the medication as intended. If that is not the case, it is our job to link patients to resources that can help, such as hospital-based financial assistance programs or grants from nonprofit organizations. Additionally, early referral to palliative care for help with optimization of pain control and management of medication side effects is critical. The American Board of Internal Medicine's Choosing Wisely campaign reminds us to not delay palliative care because a patient is pursuing disease-directed treatment.^9^ We have data, now more than a decade old, that demonstrated benefits in terms of quality of life, mood, and overall survival among patients with metastatic non–small-cell lung cancer who were randomly assigned to receive early palliative care compared with those who received standard of care.^10^ Subsequent studies have confirmed a benefit in other tumor types.^11^ Yet late referrals to palliative care are still common, occurring in up to 45% of patients according to a recent study.^12^ Finally, we should offer to provide our patients with documentation to facilitate their requests for reasonable accommodations, as mandated by the Americans with Disabilities Act, that will allow them to continue to work if that is what they choose or need to do during cancer treatment.^13^ Additional details about how we can help our patients at work can be found at Cancer and Careers,^14^ a nonprofit organization and an excellent, free, online resource that provides information for patients and clinicians about working during and after cancer treatment.

As researchers, we must continue to investigate the drivers of job loss and financial hardship among cancer survivors with the goal of identifying modifiable risk factors that can lead to interventions. For example, the data presented by Halpern et al^3^ suggest the need for an intervention to better manage pain among cancer survivors who have stopped working to promote their reintegration into the workforce. One relevant intervention could be to develop better treatments for pain, with fewer side effects, such as sedation, which can affect work ability. Another intervention could be to improve access to both pharmacologic and nonpharmacologic pain treatments through vouchers or access to free care. These approaches could be tested in a randomized trial to determine their efficacy in preventing job loss or promoting workforce re-entry.

Additional research is also needed specifically to address pain management and work among patients treated in the palliative setting. Although the National Cancer Institute considers a patient with cancer to be a survivor from the time of diagnosis until the end of life, most research on financial hardship and work outcomes focuses on patients treated with curative intent.^15^ However, issues surrounding pain, work, and finances affect patients treated in both the curative and palliative settings, as we showed in a small study at our institution.^16^ Work has important psychologic and financial benefits, and for many people, it is linked to health insurance.^17,18^ Furthermore, the advent of more effective therapies means that larger numbers of patients with incurable cancer are being treated for longer periods of time. Adequate symptom management is a critical tool to enable those who want, or need, to work to continue to do so.

Finally, it is critical to be aware that disparities on the basis of race, ethnicity, and English proficiency have been documented in pain control, access to pain medication, financial hardship, and employment outcomes in the United States.^19-21^ Disparities in level of pain were also identified by Halpern et al^3^; Hispanic and non-Hispanic Black survivors in their data set more commonly reported moderate or severe pain than non-Hispanic Whites. In response to increasing awareness of health disparities, organizations such as the American Medical Association have developed strategic plans to promote health equity.^22^ As researchers, we can contribute to these efforts by ensuring that our studies include analyses of how social determinants of health affect study outcomes and what interventions can be developed to attenuate disparities. As clinicians, we must do better to ensure that we provide equitable health care into our clinical practices, which may require ongoing education for ourselves and for all staff who engage with patients. As a society, we must work to create a more equitable health care system in which cancer survivors can access the medical care they need to thrive and continue to be valued members of our social fabric, including our workplaces, from the time of diagnosis until the end of life.
